# A Measurement Invariance Analysis of the Anxiety Scale for Autism–Adults in a Sample of Autistic and Non-Autistic Men and Women

**DOI:** 10.1007/s10803-024-06260-2

**Published:** 2024-05-14

**Authors:** Heather L. Moore, Mark Freeston, Jacqui Rodgers, Sarah Cassidy

**Affiliations:** 1 School of Psychology, Dame Margaret Barbour Building, Wallace Street, Newcastle upon Tyne, NE2 4DR UK; 2 Population Health Sciences Institute, Sir James Spence Institute, Newcastle University, Royal Victoria Infirmary, Level 3, Queen Victoria Road, Newcastle upon Tyne, NE1 4LP UK; 3 School of Psychology, University of Nottingham, University Park, Nottingham, NG7 2RD UK

**Keywords:** Autistic, Non-autistic, Anxiety, Gender, Measurement Invariance

## Abstract

The Anxiety Scale for Autism-Adults (ASA-A) captures the autistic anxiety experience, but we do not know whether it is structurally equivalent for men and women, or non-autistic people. Measurement invariance analysis considered gender and diagnostic status (342 cis-gender autistic men (*N* = 105) and women (*N* = 237), 316 cis-gender non-autistic men (*N* = 104) and women (*N* = 212)). Strict invariance was achieved between autistic men and women and between non-autistic men and women, but the ASA-A structure is quantitatively different in autistic compared to non-autistic adults. Therefore, this tool cannot be used to directly compare anxiety between these groups. Autistic women scored significantly higher on the ASA-A than autistic men and Uncertainty was the highest scoring factor for autistic participants. Future research should include alternative gender identities.

**Supplementary Information:**

The online version contains supplementary material available at 10.1007/s10803-024-06260-2.

## Introduction

Autistic people commonly experience mental health difficulties (Hollocks et al., 2018), in particular anxiety, and research has demonstrated that some aspects of anxiety differ to that of non-autistic people (Kerns et al., 2014). Gender differences also exist in rates of anxiety reported by autistic people (Uljarević et al., 2020), as well as in the general population (Baxter et al., 2013). The Anxiety Scale for Autism-Adult (ASA-A) is an anxiety measure that has been developed to capture autistic adults’ experiences of anxiety. Preliminary evaluation shows the ASA-A has good psychometric properties (Rodgers et al., 2020). Research is yet to explore whether men and women interpret the items in the ASA-A in the same way, or whether it can be used with non-autistic people, either clinically, or in research settings.

In comparison to the general population, rates of anxiety are elevated among autistic individuals (Croen et al., 2015; Nimmo-Smith et al., 2020). Among adults in the general population, a systematic review of 46 studies indicated 1-year and lifetime prevalence rates of 10.6% and 16.6%, respectively (Somers et al., 2006), while slightly higher rates of 18.1% and 28.8% were identified in a large survey of 9,282 individuals (Kessler et al., 2005a, b). In contrast, a meta-analysis using 30 studies (*N* = 26,070) published between 2000 and 2017 found 27% (CI: 17–37%) and 42% (CI: 35–50%) current and lifetime rates of any anxiety disorder in autistic people (Hollocks et al., 2018). Similar age trends have been observed, revealing increased anxiety severity, peaking in young to middle adulthood and dropping off in older age, in both autistic (Uljarević et al., 2020; Gotham et al., 2015) and general (Baxter et al., 2013, 2014) populations.

A number of studies have reported higher rates of anxiety in women than men in the general population (Baxter et al., 2013; McLean et al., 2011; Steel et al., 2014) and amongst autistic people (Murray et al., 2019; Uljarević et al., 2020). A gender by age interaction demonstrated higher increases in anxiety for women in a sample of autistic individuals and those with developmental delay (Gotham et al., 2015). However, gender differences may be due to differences in interpretation of items in the assessment measure used, differences in the symptoms experienced, or to true differences in rates of anxiety. For example, while considerable variability in findings exists, some general population studies describe a wider range of reported symptoms for women than men (Shields, 1984; Armstrong & Khawaja, 2002; Vesga-López et al., 2008; Bledsoe, 1973).

Alongside increased prevalence of anxiety, autistic people may also experience anxiety differently to non-autistic people (Renno & Wood, 2013; Kerns & Kendall, 2012; Brice et al., 2021b). The majority of research exploring the anxiety experiences of autistic people has been conducted with autistic children. Kerns et al. (2014) explored anxiety presentations in 59 autistic youth and found that 63% presented with impairing anxiety; 17% reported traditional presentations of anxiety, 15% presented with atypical anxiety, and 31% presented with both. Autism-specific, atypical presentations (in order of prevalence) included: worries about routine, novelty, and restricted interests; unusual phobias; social anxiety unrelated to fear of rejection or negative evaluation; and compulsive behaviour not motivated by attempts to relieve distress. This was supported by more recent evidence from Kerns et al. (2021). They compared autistic and non-autistic children, and found that while autistic children showed traditional presentations (21%), atypical anxiety (17%), and both (31%), non-autistic children presented only with traditional forms of anxiety (8%). In autistic adults, a number of other considerations have been identified in relation to anxiety presentations, such as IU, sensory sensitivities, negative self-evaluation as well as fear of judgement from others (Brice et al., 2021b). Strong predictors of anxiety in autism include sensory processing differences, alexithymia (difficulties with understanding and labelling emotions), and intolerance of uncertainty (IU) (South & Rodgers, 2017), all of which relate to each other (Moore et al., 2021). In response to this apparent variation in anxiety presentation, different intervention approaches are proposed (Kerns et al., 2016), and there has been some success at modifying current programmes as well as targeting some of the factors associated with autism-specific anxiety (e.g., Spek et al., 2013; Keefer et al., 2017; Rodgers et al., 2022).

Given these differences, research has focused on whether measures of anxiety developed with non-autistic populations are suitable for identifying anxiety in autistic people. Indeed, previous research has shown construct divergence and reduced sensitivity to detect anxiety across multiple anxiety measures in autistic children compared to non-autistic children (e.g., Glod et al., 2017; Kerns et al., 2015, 2017, 2021; Toscano et al., 2020; White et al., 2015) and while some tools are adequate, autism-specific measures would be better (Uljarević et al., 2018; Wigham & McConachie, 2014; Lecavalier et al., 2014; Kerns et al., 2015). Measurement issues arise for a variety of reasons. Missed and mis-diagnosis might occur because of overlap in core autism features and symptoms of anxiety (known as diagnostic overshadowing; Wood & Gadow, 2010), making differentiation between them difficult (Gjevik et al., 2011; Kerns et al., 2017), although there is evidence for statistical delineation between symptoms of each (Renno & Wood, 2013). For example, social withdrawal could be viewed as a symptom of anxiety or as social communication differences or preferences, whereas restricted and repetitive behaviours (RRB) and ritualistic behaviours associated with obsessive compulsive disorder could also be difficult to differentiate. Additional measurement difficulties arise when considering the possibility of autism-related anxiety presentations, as discussed above. Finally, measurement issues may arise from different interpretation and understanding of individual items. To illustrate, for some autistic people, separation anxiety may relate to the functional role the other person plays in helping navigate the environment (Gjevik et al., 2011; Rodgers et al., 2016), rather than the importance of a specific individual in classic separation anxiety, which may not be captured by all anxiety measures.

To address these measurement issues, autism-specific anxiety measures have been developed for autistic children (Anxiety Scale for Children-Autism Spectrum Disorder (ASC-ASD; Rodgers et al., 2016) and adults (Anxiety Scale for Autism-Adults (ASA-A; Rodgers et al., 2020). Adult anxiety research is an important area of focus given the changes in anxiety rates, with age (Gotham et al., 2015). There is a relative paucity of adult anxiety measurement research in autistic populations, particularly in comparison to the child literature. The ASA-A was developed in consultation with autistic adults and professionals working with autistic people and has good face validity and high internal consistency and test-retest reliability. Exploratory and confirmatory factor analysis indicated adequate fit with a bifactor model, comprising a General Anxiety factor and three specific factors: Uncertainty, Anxious Arousal, and Social Anxiety, with a unidimensional total score providing minimal measurement bias (Rodgers et al., 2020). However, the ASA-A is a new measure and questions remain about its measurement properties across different groups, which will clarify the potential function and limits of the ASA-A in research and clinical services. The original ASA-A development study was established with a close to 50–50 split between men and women (Rodgers et al., 2020); however, no research has yet investigated whether gender differences exist in item interpretation and degree of responding, or factor structure. This is necessary to confirm that both the measure itself, and its indicative cut-offs, are valid for both autistic men and women. A secondary consideration is whether the ASA-A is suitable as an anxiety measure for both autistic and non-autistic adults. The ASA-A was developed to provide a better representation of the anxiety experience for autistic people and a more useful clinical/research tool than those currently employed, which were developed for non-autistic populations. Thus, the ASA-A has demonstrated initial reliability and validity with autistic adults (Rodgers et al., 2020), but we do not know whether it can provide a useful equivalent for non-autistic people. This has implications not only for research where both groups are included, but also clinically, when considering whether the ASA-A could be employed more widely.

For these reasons, it is important to investigate whether responses to ASA-A items are similar for different groups (men and women, autistic and non-autistic), and so whether those items are measuring the same construct for each group (Byrne, 2004). Measurement invariance is a statistical analysis technique for testing the measurement properties of a tool across groups, by comparing the structural equivalence of the tool with increasing levels of stringency (Byrne, 2004, 2016). This will allow us to determine whether interpretation of the ASA-A is comparable (invariant) or different (non-invariant) between men and women and autistic and non-autistic people.

**Fig. 1:**
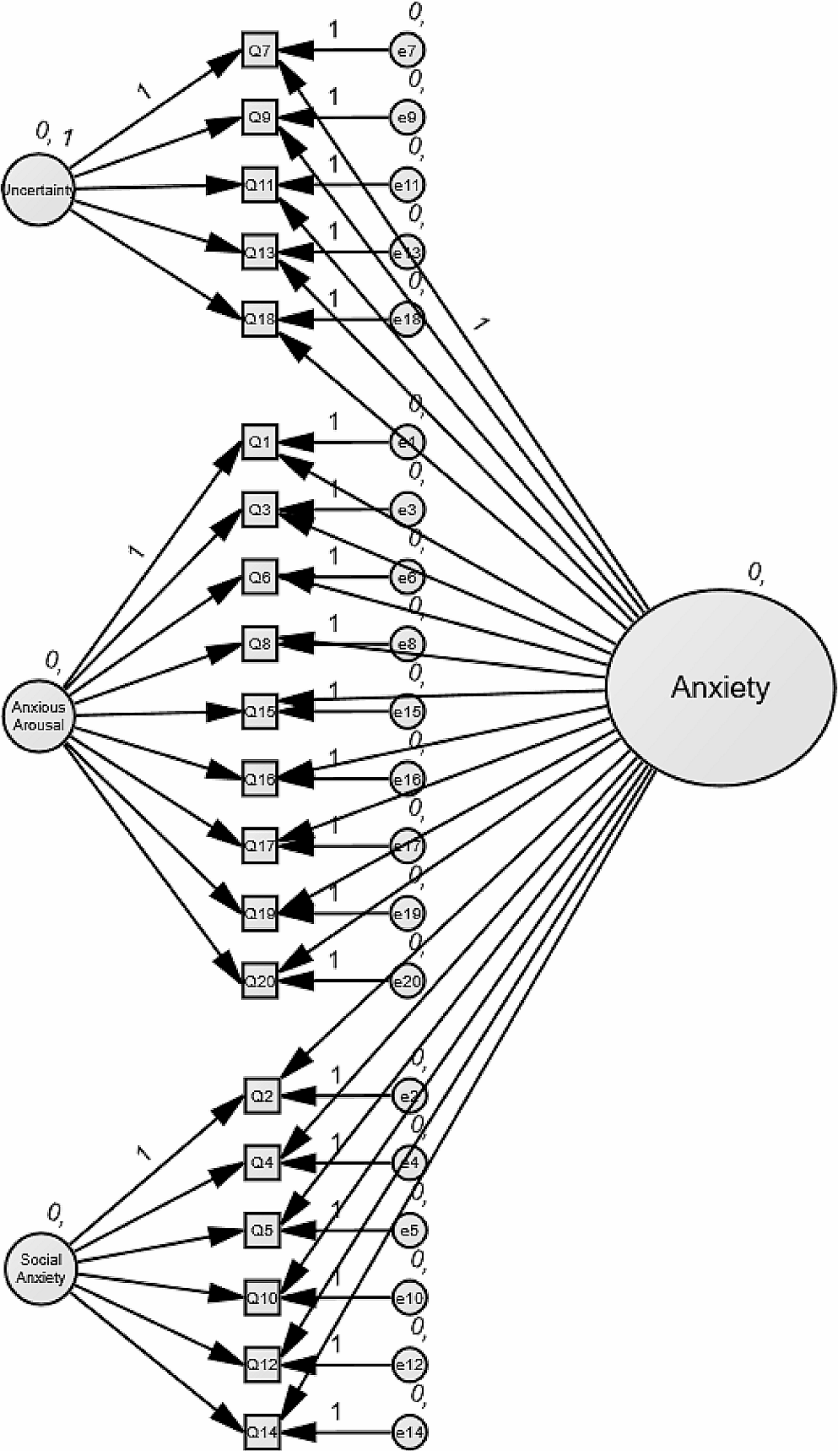
The ASA-A bifactor structure, encompassing three specific factors (Uncertainty, Anxious Arousal, and Social Anxiety), and a General Anxiety Factor

The aims of this research were to (1) Test the structural equivalence of the ASA-A bifactor model first in cis-gender autistic men and women, and second in cis-gender non-autistic men and women, (2) Test the structural equivalence of the ASA-A bifactor model between autistic and non-autistic individuals, and (3) To explore differences in anxiety across groups, where invariance has been determined. Figure 1 shows the ASA-A bifactor model.

## Methods

### Participants

We utilised data from a large online study aimed at developing a new mental health assessment tool with and for autistic adults (Cassidy et al., 2021). Participants were recruited through volunteer databases (the Autistica Discover Network and the Cambridge Autism Research Database), Twitter, and the MHAutism project website for a study aiming to adapt mental health assessment tools for autistic adults. Inclusion criteria were autistic and non-autistic people over 18 years of age and participants were informed that anyone meeting these criteria could participate, regardless of experience with mental health problems. As per requirements of measurement invariance analysis, only participants with complete ASA-A data were included in this study. Data were available for 846 participants, of which 520 identified as autistic, and 326 were non-autistic. As in previous research, this sample included some participants who self-identified as autistic (Brice et al., 2021a; Mason et al., 2018; Moore et al., 2021; Cassidy et al., 2021). However, we excluded participants who self-identified as autistic but were not seeking a diagnosis (*N* = 110), because they had significantly lower autistic traits (H(2) = 59.53, *p* < .001) than those with a formal diagnosis (*p* < .001) and those who self-identified but were seeking a diagnosis (*p* < .001), as measured by the Autism Spectrum Quotient-Short (AQ-Short (Hoekstra et al., 2011). Further comparisons between this combined group and the non-autistic group confirmed significantly higher traits in the autistic than non-autistic group, U = 101283.00, z=-20.30, *p* < .0001; Autistic mean: 22.50 (SD: 3.92), non-autistic mean: 9.33 (SD: 5.60). Due to the small sample size of non-cis-gender individuals, and the possibility that these individuals have a different experience of anxiety, we also excluded these individuals from the analyses (*N* = 75, e.g. (Sedgewick et al., 2021; Strang et al., 2021; Strauss et al., 2021). Our retained sample totalled 658 people, of which 342 identified as autistic, and 316 were non-autistic. The majority of respondents resided in the UK (autistic: 80.70%, non-autistic: 69.00%), with a spread across other countries as well (see Supplementary Table 1 for a full breakdown). The mean age of our autistic sample was 40.20 years (SD = 12.83, 18–73 years) and the mean age of our non-autistic sample was 42.17 (SD = 13.96, 19–90 years). Table 1 shows sample demographic characteristics by group.

**Table 1:** Frequency (%) of reported demographics for autistic and non-autistic participants (*N* = 658)

	Autistic Participants (*N* = 342)	Non-Autistic Participants (*N* = 316)
**Gender** ^**a**^		
Man	105	104
Woman	237	212
**Ethnicity** ^**b**^		
Asian	9 (2.6)	7 (2.2)
Black or African or Caribbean	4 (1.2)	5 (1.6)
Hispanic or Latinx	8 (2.3)	6 (1.9)
Middle Eastern or Arab	1 (0.3)	2 (0.6)
White or Caucasian	312 (91.2)	291 (92.1)
Other Ethnic Group	13 (3.8)	12 (3.8)
Prefer Not to Answer	3 (0.9)	2 (0.6)
**Living Situation**		
Living independently	93 (27.2)	56 (17.7)
Living with a partner and/or dependent(s)	145 (42.4)	215 (68.0)
Living with flatmate(s)	9 (2.6)	17 (5.4)
Living with friend(s)	5 (1.5)	4 (1.3)
Living with parents	70 (20.5)	22 (7.0)
Living in supported accommodation	4 (1.2)	0 (0.0)
Living with a carer	2 (0.6)	0 (0.0)
Other	14 (4.1)	2 (0.6)
**Employment Status** ^**b**^		
Full time	101 (29.5)	177 (56.0)
Part time	73 (21.3)	64 (20.3)
Volunteer full time	1 (0.3)	1 (0.3)
Volunteer part time	43 (12.6)	15 (4.7)
Student full time	38 (11.1)	33 (10.4)
Student part time	33 (9.6)	15 (4.7)
Retired	13 (3.8)	26 (8.2)
Looking for work	18 (5.3)	6 (1.9)
Not looking for work	18 (5.3)	7 (2.2)
Unable to work	93 (27.2)	9 (2.8)
**Education Setting** ^**b**^		
Home schooling	8 (2.3)	3 (0.9)
Mainstream	329 (96.2)	305 (96.5)
Special school	10 (2.9)	4 (1.3)
Other	7 (2.0)	8 (2.5)
**Highest Qualification** ^**c**^		
None	7 (2.0)	1 (0.3)
NVQ Level 1	2 (0.6)	0 (0.0)
NVQ Level 2	8 (2.3)	8 (2.5)
NVQ Level 3	13 (3.8)	14 (4.4)
GCSE/O Level	31 (9.1)	8 (2.5)
A Level/Higher	42 (12.3)	15 (4.7)
Undergraduate Degree	107 (31.3)	99 (31.3)
Higher Degree	104 (30.4)	160 (50.6)
Other	27 (7.9)	11 (3.5)
**Diagnosed Developmental Condition**		
Yes	95 (27.8)	28 (8.9)
No	247 (72.2)	288 (91.1)
**Developmental Condition** ^**b**^		
Dyspraxia	30 (8.8)	3 (0.9)
Learning disabilities	5 (1.5)	1 (0.3)
Learning difficulties	2 (0.6)	0 (0.0)
Dyscalculia	8 (2.3)	0 (0.0)
Dyslexia	28 (8.2)	12 (3.8)
ADHD	42 (12.3)	14 (4.4)
Developmental delay	5 (1.5)	1 (0.3)
Other	12 (3.5)	0 (0.0)
**Diagnosed Mental Health Condition**		
Yes	241 (70.5)	93 (29.4)
No	101 (29.5)	223 (70.6)
**Mental Health Condition** ^**b**^		
Depression	172 (50.3)	75 (23.7)
Anxiety	201 (58.8)	58 (18.4)
Obsessive Compulsive Disorder (OCD)	29 (8.5)	6 (1.9)
Bipolar disorder	14 (4.1)	3 (0.9)
Personality disorder	26 (7.6)	6 (1.9)
Schizophrenia	0 (0.0)	1 (0.3)
Post Traumatic Stress Disorder (PTSD)	52 (15.2)	10 (3.2)
Tourette’s Syndrome / Tic Disorder	4 (1.2)	0 (0.0)
Anorexia	15 (4.4)	2 (0.6)
Bulimia	7 (2.0)	3 (0.9)
Other	31 (9.1)	3 (0.9)

*Note*: NVQ: National Vocational Qualification; GSCE: General Certificate of Secondary Education; O Level: General Certificate of Education Ordinary Level; A Level: Advanced Level

^a^Cis-gender; gender identity matches birth assignment

^b^Multiple responses possible

^c^1 missing from autistic group

A favourable ethical opinion was obtained from the School of Psychology ethics committee at the University of Nottingham (F1074). All participants provided informed consent.

### Measures

This study is part of a wider research project to adapt tools with and for autistic people (Cassidy et al., 2021)^1^. Measures used here were:

#### Autism Spectrum Quotient-Short (AQ-Short; Hoekstra et al., 2011)

The AQ-Short (Hoekstra et al., 2011) is a condensed version of the 50-item Autism Spectrum Quotient (AQ; Baron-Cohen et al., 2001), a self-report measure of autism traits that encompasses ‘social behavioural difficulties’ and ‘a fascination for numbers/patterns’. The AQ-Short consists of 28 items on a four-point Likert scale, ranging from 1 (*definitely agree*) to 4 (*definitely disagree*). Total scores ranged from 28–112 and scores of > 65 provides a useful threshold when screening to indicate further assessment may be warranted (sensitivity: .97 − 1, specificity: .82-.94 to discriminate autistic and non-autistic individuals; Hoekstra et al., 2011; Lugo-Marín et al., 2019), although lower scores have also been found (Sizoo et al., 2015). Comparison between autistic men and women also supports the same two-factor structure, as well as equivalent interpretation of items through formal measurement invariance testing, suggesting it is suitable for use with both groups (Grove et al., 2016).

#### Anxiety Scale for Autism-Adults (ASA-A; Rodgers et al., 2020)

The ASA-A is a 20-item, self-report measure of anxiety developed specifically for autistic adults. It consists of a General Anxiety factor as well as three group factors pertaining to Uncertainty (U), Anxious Arousal (AA), and Social Anxiety (SA) (see Fig. 1). Scores range from 0 (*never*) to 3 (*always*) with the total ranging from 0 to 60 and a total score of ≥ 28 indicating the presence of significant levels of anxiety. Internal consistency is excellent for the general factor and group factors (General: 0.90; SA: 0.85, AA: 0.85, U: 0.83; Rodgers et al., 2020). Test-retest reliability is excellent (0.82), and convergent (Hospital Anxiety and Depression Scale (HADS)-Anxiety (Zigmond & Snaith, 1983): 0.70) and divergent (HADS-Depression: 0.47) validity are robust (Rodgers et al., 2020). In our sample, Cronbach’s alpha was 0.93 for the autistic participants, and 0.94 for the non-autistic participants, indicating excellent reliability.

### Procedure

Participants completed the survey online through Qualtrics. Participants first provided demographic information, including self-report of autism status, and diagnosed developmental and mental health conditions. They then completed the AQ-Short and ASA-A, alongside other measures relevant to the wider study.

### Statistical Analyses

Data were prepared and analysed using SPSS Statistics Version 25 (IBM Corp, 2017) and SPSS AMOS Version 27 (Arbuckle, 2020).

Item level scores and total scores were used for the measures. To calculate total scores, item scores were summed for the AQ-Short and the ASA-A. We used AQ-Short scores to determine whether we could combine our formally diagnosed and self-identifying autistic groups into one group for measurement invariance analysis (see Participants). Participants could choose to skip sections of the survey and were able to decline to answer questions on any measure. Only those with complete ASA-A data were included in these analyses (see Cassidy et al., 2021 for further details of missing data from the whole sample). Missing value analysis indicated that 1.2% of AQ-Short total scores were missing.

#### Measurement Invariance

We conducted three rounds of measurement invariance analysis in SPSS AMOS, to answer the following questions:

1. Is the ASA-A measurement invariant across cis-gender autistic men and women?
2. Is the ASA-A measurement invariant across cis-gender non-autistic men and women?
3. Is the ASA-A measurement invariant across autistic and non-autistic individuals?

Sample size was acceptable for comparing two groups, but greater sample size would be beneficial for comparing all four groups in one combined analysis (autistic men, autistic women, non-autistic men, non-autistic women), due to the increased model complexity (Kline, 2016).

The following methodology was applied to each question. First, confirmatory factor analysis was applied to each group, to confirm adequate fit to the published bifactor model (Fig. 1). The χ2 statistic is likely to be affected by larger sample sizes (Stevens, 2012), so was not used in these analyses. Hu & Bentler (1998, 1999) recommend a combination of absolute fit, incremental fit, and residual-based indices of model fit:

- Absolute measures of fit tend to consider how close the observed model is to a perfect fit (or zero), so lower scores indicate better fit. Here, we use the χ2/df ratio and Root Mean Square Error of Approximation (RMSEA). In addition to these, the p of Close Fit (PCLOSE) measures the probability that the RMSEA is less than or equal to 0.05.
- Incremental fit indices compare the observed data to a worse fitting model and higher scores indicate better fit. Measures included here are the Comparative Fit Index (CFI) and Tucker-Lewis Index (TLI).
- Residual-based measures (such as the Standardized Root Mean Square Residual (SRMR) used here) examine the standardised discrepancy between observed correlations and hypothesised correlations, and lower scores indicate better fit.

Table 2 shows the recommended criteria for model fit on each measure.

**Table 2:** Indices of model fit for confirmatory factor analysis

Index of Fit	Thresholds for Goodness of Fit		
	Poor	Acceptable/Good	Excellent
χ2*/df* ratio (Bryant & Yarnold, 1995)		< 5	< 3
RMSEA (Browne & Cudeck, 1993; Hu & Bentler, 1999)	0.08-0.1	< 0.06	< 0.05
PCLOSE		> 0.05	
CFI (Browne & Cudeck, 1993)		> 0.90	> 0.95
TLI (Brown, 2015)		> 0.90	> 0.95
SRMR (Hu & Bentler, 1999)		< 0.08*	

*Note*: CFI: Comparative Fit Index; TLI: Tucker-Lewis Index; RMSEA: Root Mean Square of Approximation; PCLOSE: p of Close Fit; SRMR: Standardized Root Mean Square Residual

* <0.09 in combination with an RMSEA of < 0.06 (Hu & Bentler, 1999)

Next, a series of nested models were tested to compare the structural equivalence between groups. Each model applied increasing constraints, to assess increasingly strict levels of measurement invariance between the groups (Byrne, 2004).

1. Configural invariance has no equality constraints, and assesses whether the items measure the same latent variables (i.e., the ASA-A bifactor model) in both groups. This model simply tests whether the same number of factors is relevant, and whether the same items are salient to each factor.
2. Metric invariance constrains the factor loadings to be equal between the groups and assesses whether the strength of the relationship between items and the factor are the same for both groups. If metric non-invariance occurs, then the groups interpret the items in different ways, so cannot be compared.
3. Scalar invariance constrains the intercepts of the items to be equal between groups and assesses whether total scores on the scale consist of similar item scores in each group. At this point, the groups can be compared using this measure. If scalar non-invariance occurs, then this suggests that the groups use the scale in different ways, and so different cut-offs can be developed for each group, by using ROC analyses (Meredith, 1993; Steinmetz, 2013).
4. Residual invariance constrains error terms and error co-variances to be equal between the groups (i.e. model item unique variance between the groups). It assesses whether the scale items measure the latent constructs with the same amount of measurement error. If residual non-invariance occurs, it is still acceptable to compare groups using this measure (Meredith, 1993; Steinmetz, 2013).

Configural invariance was first assessed for the best model fit. If this was achieved, then each subsequent model was assessed for degradation in model fit compared to the configural model, using the following criteria:

- A significant change in chi-square (Gaskin, 2016).
- A decrease in RMSEA of > 0.015 (Chen, 2007).
- A decrease in CFI of > 0.01 (Cheung & Rensvold, 2002).

If invariance was not achieved at a particular step, then further, stricter levels of invariance were not tested. At this point, sources of non-invariance were explored by constraining parts of the model one at a time and assessing degradation in model fit. We employed stricter tests of measurement invariance, by adding newly constrained items to those that were previously shown to be invariant, and so building the number of constraints up (Byrne, 2004).

#### Reliability of the General and Specific ASA-A Factors

Before conducting any group comparisons, reliability of the General Anxiety factors and three specific factors (U, AA, and SA) were tested to determine whether a unidimensional or multidimensional model was most appropriate, and whether total and specific factor scores can be used from the measure. Measures of reliability included:

- Explained Common Variance (ECV): Proportion of common variance explained by that factor. For specific factors, this is the strength of a specific factor relative to all explained variance for the items that load on to that factor only. It gives an indication of how unidimensional the instrument is, and an ECV of ≥ 0.85 indicates sufficient unidimensionality to warrant a one-factor model (Stucky & Edelen, 2014; Stucky et al., 2014).
- Percent of Uncontaminated Correlations (PUC): Measured for the general factor, a higher PUC suggests less bias in the structural coefficients, giving support to a unidimensional model. Guidance varies regarding the PUC, but two criteria for lower relative bias and unidimensionality of the construct are:
  - When ECV is > 0.70 and PUC is > 0.70 (Rodriguez et al., 2016).
  - When PUC is < 0.80, general factor ECV is > 0.60 and general factor OmegaH is > 0.70 (Reise et al., 2013b).
- Omega Hierarchical (ωH): ω is the proportion of total variance that can be attributed to all common factors, whereas ωH is the proportion of total variance attributed to the general (or specific) factor after accounting for specific (or general) factors. A minimum of 0.50, and preferably > 0.75, indicates that the factor score reflects the target dimension (i.e. specific or general factor; Reise et al., 2013a).
- Percentage of Reliable Variance (PRV): Is calculated using ωH/ω, and indicates whether the factor in question contributes added value beyond other factors with shared variance (e.g., specific after accounting for variance accounted for by the general factor, and vice versa). No agreed guidelines currently exist, but Reise et al.’s (2013a) guidelines of > 0.50 (> 0.75 better) have been applied here.

#### Group Comparisons

Where strict measurement invariance was achieved, and guided by the bifactor structure reliability information, appropriate group comparisons were explored. Where specific factor scores were compared, they were first converted to Z-scores so that they were on the same scale. As the data were not normally distributed, non-parametric alternatives were used where possible. There is no nonparametric equivalent to a mixed ANOVA, but ANOVAs are considered robust to violations of normality (e.g., Blanca et al., 2017).

## Results

### Confirmatory Factor Analysis

Table 3 shows model fit results for the CFA for autistic individuals, non-autistic individuals, and a combined sample of autistic and non-autistic individuals. For the autistic sample, two error terms were co-varied to achieve the best model fit (errors for items 15&19, 8&16). For the non-autistic sample, three error terms were co-varied to achieve the best model fit (errors for items 15&19, 10&14, 3&6). In the combined sample, five error terms were co-varied to achieve the best model fit (errors for items 15&19, 3&6, 4&5, 5&12, 6&17). CFA indicated good model fit for each group; therefore, we proceeded to measurement invariance analysis.

**Table 3:** Indices of model fit for confirmatory factor analysis and measurement invariance, as well as measures of model degradation for measurement invariance analyses, for autistic and non-autistic adult groups

	Model Fit Indices						Indices of Degradation in Model Fit				
	χ2*/df* ratio	RMSEA	PCLOSE	CFI	TLI	SRMR	∆χ2	∆DF	∆p	∆RMSEA	∆CFI
Cis-Gender Autistic Men and Women											
CFA^a^	1.808	0.049	0.581	0.965	0.955	0.047					
Configural	1.713	0.046	0.848	0.937	0.921	0.106					
Metric	1.522	0.039	0.997	0.948	0.942	0.083	0.113	38	1.0000	0.007	-0.011
Scalar	1.502	0.038	0.999	0.947	0.944	0.083	23.545	58	1.0000	0.008	-0.010
Residual	1.541	0.040	0.997	0.940	0.940	0.089	68.494	78	0.7705	0.006	-0.003
Cis-Gender Non-Autistic Men and Women											
CFA^b^	1.851	0.052	0.356	0.966	0.957	0.042					
Configural	1.781	0.050	0.503	0.937	0.921	0.055					
Metric	1.573	0.043	0.962	0.948	0.942	0.066	3.201	38	1.0000	0.007	-0.011
Scalar	1.555	0.042	0.976	0.947	0.944	0.066	21.839	58	1.0000	0.008	-0.010
Residual	1.579	0.043	0.966	0.942	0.942	0.065	61.902	78	1.0000	0.007	-0.005
Autistic and Non-Autistic Individuals											
CFA^c^	2.215	0.043	0.966	0.983	0.978	0.024					
Configural	2.008	0.039	1.000	0.958	0.947	0.040					
Configural (error 15&19)	1.803	0.035	1.000	0.967	0.957	0.040					
Configural (+ error 3&6)	1.707	0.033	1.000	0.971	0.962	0.039					
Metric*	1.925	0.038	1.000	0.957	0.951	0.061	646.864	40	< 0.0001	− 0.0005	-0.014

RMSEA: Root Mean Square Error of Approximation; PCLOSE: p of Close Fit; CFI: Comparative Fit Index; TLI: Tucker-Lewis Index; SRMR: Standardized Root Mean Square Residual; ∆: Change

(a) Model parameters for CFA with error terms for items 15&19 and 3&6 co-varied. (b) Model parameters for CFA with error terms for items 15&19, 10&14, and 3&6 co-varied. (c) Model parameters for CFA with error terms for items 15&19, 3&6, 4&5, 5&12, and 6&17 co-varied

* Significant degradation of fit from the configural model

### Measurement Invariance

Table 3 shows the measurement invariance model fit and degradation of fit results for all three questions. Two error terms (items 15&19, 3&6) were co-varied to achieve the best configural model fit for the analysis comparing autistic and non-autistic responses to the ASA-A. Configural invariance met criteria for good model fit for each analysis. For the measurement invariance analyses between cis-gender autistic men and women, and cis-gender non-autistic men and women, no significant degradation of model fit was identified for each subsequent test of invariance. As such, residual invariance was achieved, and the final models showed good model fit overall (Table 3).

As residual measurement invariance was achieved between cis-gender men and women for both autistic and non-autistic groups, samples were combined to compare responses of autistic and non-autistic individuals. To achieve the strongest model fit, two error terms were co-varied (Table 3), and configural invariance was achieved. However, when metric invariance was assessed, the model fit remained good, but there was significant degradation in model fit from the configural model. As such, no further steps were tested. Instead, item level sources of metric non-invariance were explored. Supplementary Table 2 shows all constraints tested in a stepwise fashion. First, each factor was tested individually, which showed that all items from the Anxious Arousal (AA) factor were invariant between the groups, but Uncertainty (U) was not, and neither were the Social Anxiety (SA) and General Anxiety factors, when added to the constrained AA factor. Next, individual items from the U factor, followed by the SA factor, and then the General Anxiety factor were added one at a time to the constrained AA factor. Any invariant items remained constrained as additional items were added. The final constrained model showed that all items, except question 13 from the U factor, and question 10 from the SA factor, were invariant between the groups. However, only nine of 20 items were invariant for the General Anxiety factor (questions 9, 11, 18, 1, 3, 8, 15, 17, and 20). The final model showed good fit overall (Supplementary Table 2).

### Reliability of the ASA-A General and Specific Factors

Table 4 reports measures of reliability of the general and specific factors for use when calculating scores. ECV and PUC scores for both the autistic and non-autistic samples support a multidimensional construct, but with most variance predicted by the General Anxiety factor, and specific factors explain very little common variance beyond that accounted for by the General Anxiety factor.

**Table 4:** Model reliability estimates for the general anxiety factor and specific factors of uncertainty, anxious arousal, and social anxiety

	ECV	PUC	ω	ωH	PRV
Autistic Cis-Gender Men and Women					
General Anxiety	0.54	0.68	0.95	0.77	0.81
Uncertainty	0.24		1.08	0.07	0.06
Anxious Arousal	0.21		1.00	0.26	0.26
Social Anxiety	0.23		1.06	0.30	0.28
Non-Autistic Cis-Gender Men and Women					
General Anxiety	0.65	0.68	0.96	0.85	0.88
Uncertainty	0.20		1.07	0.05	0.05
Anxious Arousal	0.19		1.00	0.19	0.19
Social Anxiety	0.18		1.05	0.22	0.22

### Group Comparisons

As strict measurement invariance was identified between cis-gender autistic men and women, and between cis-gender non-autistic men and women, we were able to compare ASA-A total scores between these groups. As measurement invariance was not achieved between autistic and non-autistic groups, we were not able to compare scores between these groups. Table 5 shows ASA-A total scores for each group. Mann Whitney U tests were used to compare total scores. Autistic women scored significantly higher on the ASA-A than autistic men (U = 9872.50, z=-3.05, *p* = .0023). Using the published cut-off of ≥ 28, 80.7% of autistic individuals showed the presence of significant levels of anxiety (69.5% of men and 85.7% of women). In the non-autistic sample, there was no significant difference in ASA-A total scores between men and women (U = 10082.00, z=-1.24, *p* = .2168). Given non-invariance between autistic and non-autistic samples, we do not know whether published cut-offs are suitable for non-autistic individuals, so these were not applied.

**Table 5:** Descriptive statistics on the ASA-A total score, split by autism diagnosis and gender

Gender	N	Mean	SD	Min	Max
Autistic Cis-Gender Men and Women					
Men	105	34.84	10.66	10.00	59.00
Women	237	38.62	10.94	4.00	59.00
All	342	37.46	10.98	4.00	59.00
Non-Autistic Cis-Gender Men and Women					
Men	104	17.87	10.55	0.00	54.00
Women	212	19.65	11.41	2.00	55.00
All	316	19.06	11.15	0.00	55.00

**Fig. 2:**
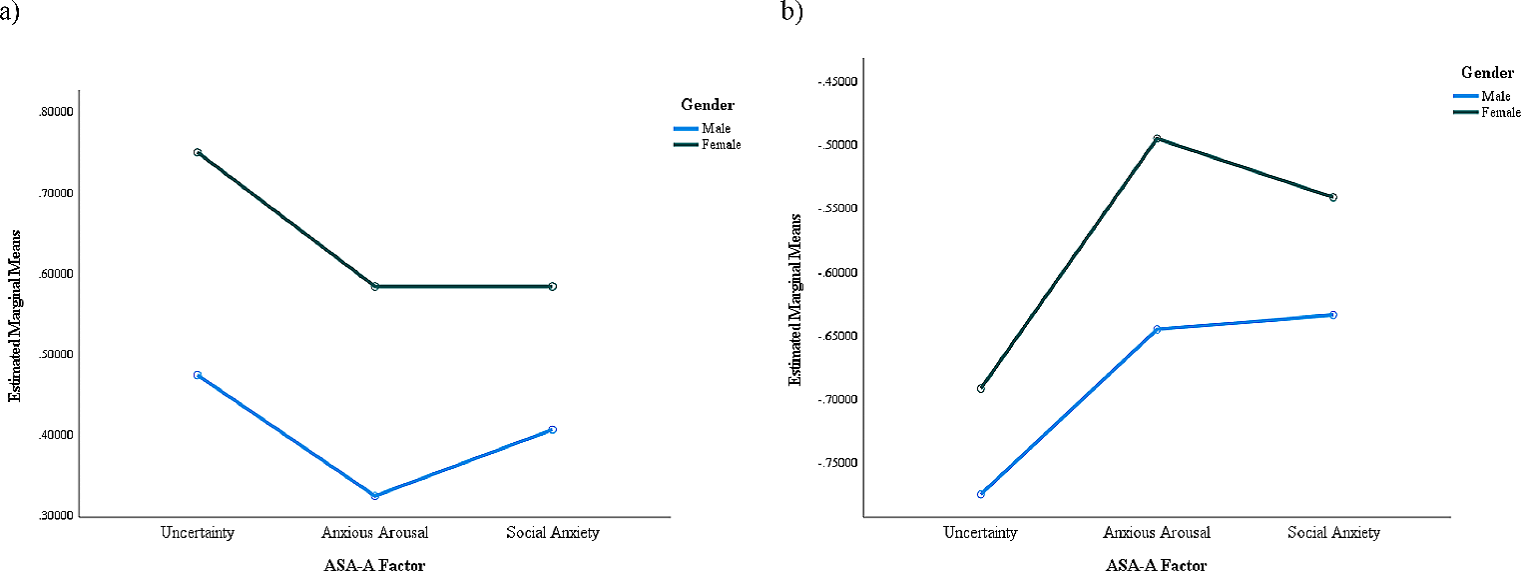
**a**, **b** Mean Z-scores on the Uncertainty, Anxious Arousal, and Social Anxiety factors of the ASA-A, for autistic and non-autistic adults, split by gender

While reliability of the bifactor structure indicates that a unidimensional score is most appropriate, conceptually, it is useful to understand the relative score for each specific factor in the anxiety presentation within each group by gender (but not between groups). Converting subscale total scores to Z-scores, we ran a mixed ANOVA, with gender as the between-subjects variable and ASA-A specific factor score as the within-subjects variable, for both autistic and non-autistic groups. Figure 2a and b shows the breakdown of factor scores for each group, split by gender.

A mixed ANOVA demonstrated significant differences between factor scores for autistic participants (F(1.88, 638.22) = 6.57, *p* = .0018; Greenhouse-Geisser correction), but no ASA-A factor X gender interaction (F(1.88, 638.22) = 0.68, *p* = .4976; Greenhouse-Geisser correction; see Fig. 2a). Pairwise comparisons using Bonferroni adjustment for multiple comparisons showed that scores for U were significantly higher than AA (*p* = .0007) and SA (*p* = .0167) but AA and SA scores did not differ significantly (*p* = 1.0000). For the between-subjects effect of gender, significantly higher scores for women were confirmed (F(1, 340) = 9.21, *p* = .0025. Interestingly, non-autistic cis-gender men and women showed a different pattern of scores on the ASA-A factors to that of autistic participants (see Fig. 2b). A mixed ANOVA demonstrated significant differences between factor scores (F(1.91, 598.64) = 10.60, *p* < .0001; Greenhouse-Geisser correction), but no ASA-A factor X gender interaction (F(1.91, 598.64) = 0.44, *p* = .6380; Greenhouse-Geisser correction; see Fig. 2b) for non-autistic participants. Pairwise comparisons using Bonferroni adjustment for multiple comparisons showed that U scores were significantly lower than both AA (*p* < .0001) and SA (*p* = .0011), but AA and SA scores did not differ significantly (*p* = 1.0000). Replicating the results with the total scores, there were no significant differences between men and women (F(1, 314) = 1.64, *p* = .2015).

## Discussion

To date, this is the first study to investigate the structural equivalence of the ASA-A for autistic and non-autistic adults, comparing cis-gender men and women within groups, as well as comparing across groups. Strict residual measurement invariance was achieved across gender within groups alongside good model fit, but metric non-invariance was found between autistic and non-autistic participants. For both groups, a multidimensional (bifactor) structure was supported, but specific factors did not add much beyond their contribution to the General Anxiety factor. Cis-gender autistic women demonstrated significantly higher levels of anxiety than cis-gender autistic men, but there were no gender differences for non-autistic participants. The relative contribution of specific factors to the General Anxiety factor varied between groups, with Uncertainty contributing most for autistic participants, and least for non-autistic participants.

### Structural Equivalence of the Bifactor ASA-A

Importantly, our findings show strict measurement invariance between autistic cis-gender men and women, demonstrating that not only do autistic men and women interpret the items in the same way, but they also use the rating scale in the same way, and there is likely to be the same amount of measurement error between the groups. Very little work has investigated gender differences in response to autism-specific screening measures for mental health and other difficulties to date. Our results mirror evidence of scalar invariance from the Suicidal Behaviours Questionnaire-Autism Spectrum Conditions (SBQ-ASC; Cassidy et al. (2021), and residual invariance on the Camouflaging Autistic Traits Questionnaire (CAT-Q; Hull et al., 2019) between genders. Our findings have important implications both clinically and within a research setting. Clinically, the indicative cut-offs for anxiety for autistic adults reported by Rodgers et al. (2020) can be use with both cis-gender autistic men and women, although further validation of the cut-off is required. In a research setting, it is also appropriate to use the ASA-A to compare cis-gender men and women. Interestingly, non-autistic men and women also achieved strict measurement invariance on the ASA-A, suggesting that they also interpret and use this measure in the same way as each other. In contrast to autistic populations, there is a wealth of literature demonstrating gender invariance on anxiety (and other mental health) measures in non-autistic populations (e.g., Bunnell et al., 2013; Ebesutani et al., 2014; Fong & Ho, 2014; Tindall et al., 2021). This is unlikely to be the most suitable measure for non-autistic individuals though.

Comparing autistic and non-autistic individuals, metric invariance was not achieved, suggesting that ASA-A items are interpreted differently by autistic and non-autistic individuals. Comparisons of the structural equivalence of other measures for autistic and non-autistic individuals have demonstrated varying levels of invariance, ranging from strict measurement invariance on the CAT-Q (Hull et al., 2019), to scalar non-invariance on the AQ-Short (Murray et al., 2014), and metric non-invariance on the SBQ-R (Cassidy et al., 2020). It is possible that some topics are represented in the same way to a greater and lesser extent for autistic and non-autistic people, and further research needs to determine whether the source of non-invariance stems from item or construct understanding differences in each case.

Drilling into the source of non-invariance on the ASA-A, most items loading on to the three-factor model were metric invariant, with a few exceptions, and indeed, the Anxious Arousal factor was fully metric invariant. One example of non-invariant specific factor items was question 13 (“I need to be prepared before things can happen”; Uncertainty). IU is defined as a negative reaction to unforeseen/unpredictable situations and events (Freeston et al., 1994) and the Intolerance of Uncertainty Scale (Carleton et al., 2007) focuses more on the negative impact that IU has (e.g. “Unforeseen events upset me greatly”, “The smallest doubt can stop me from acting”). This item focuses more on a need to plan, and so may reflect autism-related element of uncertainty-related anxiety.

While most specific factor items were metric invariant, more than 50% of items loading on to the General Anxiety factor were metric non-invariant. Of these, all Social Anxiety items were non-invariant for the General Anxiety factor. These items focus on performance judgement, which may reflect the social communication difficulties and associated stigma that autistic people experience. Social anxiety is more prevalent in autistic than non-autistic individuals (Maddox & White, 2015; Nice, 2013; Spain et al., 2016) and may represent a core feature of anxiety specific to autistic people. Thus, it appears that while there is a similar interpretation of items at the specific factor level, when integrated, these items do not represent the same anxiety experience for non-autistic people, corroborating the evidence of important differences in mental health experiences for autistic and non-autistic people (Kerns et al., 2014; Renno & Wood, 2013). As such, the ASA-A total score cannot be used to compare these two groups.

These results suggest that the ASA-A total score in its current form cannot be used as a measure of anxiety for non-autistic people, and that an individualised approach to assessing anxiety in autistic and non-autistic groups should be taken, using measures tailored to capture the mental health experiences of different groups. Of course, this also presents problems for comparing anxiety in autistic and non-autistic groups in a research setting. The near metric invariant three-factor solution could be explored in future research to determine whether a reduction of items could lead to metric invariance without sacrificing the sensitivity and specificity of the ASA-A in autistic samples. This could lead to a comparable three-factor solution to use for research purposes. The limits of using the specific factor scores must be acknowledged though. For both groups, a multidimensional (bifactor) structure was supported, but items on the specific factors were mostly capturing variance in the General Anxiety factor rather than unique variance, supporting the currently employed unidimensional total score over use of the specific factor scores. As such, it is unlikely that the ASA-A is an appropriate tool for non-autistic individuals and more accurate results might be obtained by using the optimal measure for each group and then converting them to the same scale for group comparisons.

### Group Differences on the ASA-A

We explored group differences in ASA-A scores where strict invariance was achieved, and found that cis-gender autistic women demonstrated significantly higher levels of anxiety than cis-gender autistic men, in keeping with previous research (Murray et al., 2019; Uljarević et al., 2020), but in contrast to the evidence base for non-autistic groups, there were no gender differences for non-autistic participants in this study (Baxter et al., 2013; McLean et al., 2011; Steel et al., 2014). The rates of anxiety in our autistic sample were substantially higher than has been previously reported using estimates from a large pooled sample (Hollocks et al., 2018), and somewhat higher than the number with a diagnosed anxiety disorder in our sample (58.8%). Higher rates of anxiety might be identified when more appropriate screening measures for autistic people are used, and using the ASA-A might elevate the pooled estimates identified by Hollocks et al. (2018). Alternatively, further validation of the indicative cut-off reported by Rodgers et al. (2020) might be warranted through comparison against a structured clinical interview. For example, the Anxiety and Related Disorders Interview Schedule for DSM-5 (ADIS-5; Brown & Barlow, 2014) is a commonly used anxiety assessment and has been used to describe and diagnose non-standard anxiety presentations in autistic adults (Parr et al., 2020). However, as the ADIS-5 has not been developed or validated for autism, the more recently developed Personalised Anxiety Interview Schedule–Autism (PAIS-A; Brice et al., 2021b) might be used in conjunction with the ADIS-5 or other diagnostic interviews, to confirm whether the sensitivity and specificity of this indicative cut-off holds in an independent sample of autistic adults. Self-selection based on interest in, and experience of, mental health difficulties is also likely (Rubenstein & Furnier, 2021). These different explanations make it more difficult to determine true rates of anxiety in this population and large-scale community sampling would help to answer this question in future. Cut-offs were not applied to the non-autistic sample, given that the identified metric non-invariance raises questions of whether these cut-offs are accurate for non-autistic individuals.

While we could not compare autistic and non-autistic participants directly, we did look at the relative scores of different facets of anxiety measured by the ASA-A for each group. Uncertainty made up the largest component for autistic participants, but the smallest component for non-autistic participants, further supporting differences in the experience of anxiety in each group. IU is well established as a transdiagnostic mechanism of anxiety in autistic (Jenkinson et al., 2020) and non-autistic (Buhr & Dugas, 2009; Carleton et al., 2012; McEvoy et al., 2019) populations and is increasingly recognised as a particular area of difficulty for autistic individuals. The higher relative contribution of Uncertainty compared to other facets of anxiety seen in our autistic sample supports this and has important clinical implications about where to focus anxiety interventions in this group. Currently, interventions may use a model employed with non-autistic people, or perhaps adapt a current intervention to make it more accessible to autistic individuals, with some recognising the role of IU in outcomes (Keefer et al., 2017; Spek et al., 2013). Our results, however, add to a growing body of evidence that suggest that interventions need to be designed to meet the specific needs of autistic people, to have the best outcomes. A recent RCT demonstrated improved anxiety treatment outcomes for autistic children when using a tailored CBT programme over standard CBT (Wood et al., 2020), while another study has demonstrated feasibility of a parent-based intervention designed specifically to address difficulties with uncertain situations experienced by autistic children (Rodgers et al., 2022). One adult intervention is currently being trialled that the authors are aware of; this provides an individualised intervention package based on the needs of the individual, including IU (Parr et al., 2020). With interventions focusing on autism-specific anxiety, the ASA-A may prove a useful tool for assessing outcomes.

### Strengths and Limitations

One of the strengths of our study is the large sample size, including both autistic and non-autistic cis-gender men and women, and our analyses were well powered. In both groups, we had more women than men, which fits with response rates in survey research generally (Sax et al., 2008). While survey studies with autistic individuals show a stronger bias towards men (e.g., Gelbar et al., 2014), women are more likely to participate in mental health research (Crisp & Griffiths, 2014; Woodall et al., 2010), in alignment with our own gender demographics. Unfortunately, our sample did not include enough participants with alternative gender identities to be able to include these individuals in our measurement invariance analysis. People who identify as autistic have increased likelihood of also identifying with other minority groups relating to gender and sexual identity (Dewinter et al., 2017; Glidden et al., 2016; Van Der Miesen et al., 2016). In both autistic and non-autistic individuals, gender diversity is associated with increased mental health difficulties, and there is emerging evidence that autism and gender diversity combine to increase risk of mental health difficulties (Sedgewick et al., 2021; Strang et al., 2021; Strauss et al., 2021). However, very little is known about whether gender diversity impacts interpretation of mental health measures. Thus, there is a clear need to conduct research with people with alternative gender identities, in order to understand more about their experience of anxiety, and whether the ASA-A adequately captures this.

Another area that might influence interpretation of measures is culture. No research that the authors are aware of has explored this in relation to mental health in autism, but there is some evidence of cultural differences in depression in general population samples (Lee et al., 2014; Di Florio et al., 2017). One study that investigated measurement invariance across countries identified small group differences in the Patient Health Questionnaire (PHQ-9; Kroenke et al., 2001) and Generalized Anxiety Disorder scale (GAD-7; Spitzer et al., 2006), though they argue these were unlikely to affect interpretation of results. With 91–92% of our sample identifying as White or Caucasian, and 81% (autistic) and 69% (non-autistic) residing in the UK, our sample was underpowered to explore any differences based on culture, but future research should explore how culture interacts with autism status and mental health.

It is important to note that we used self-reported autism status, diagnosed developmental and mental health conditions, and educational qualification status to characterise our sample. While this may limit the accuracy of our sample characterisation, this information was not used in any of the primary analyses, and as such, should not affect our results or conclusions. Finally, very few participants in either group reported a diagnosed intellectual disability or other developmental condition, and the majority have some qualifications (61.7% of autistic people and 81.9% of non-autistic people reported an undergraduate or higher degree). Work with autistic children has demonstrated variation in anxiety presentation, as well as sensitivity of anxiety measures, based on intellectual ability (Kerns et al., 2021). While the ASA-A is self-report, and so a certain level of ability is pre-requisite to complete this measure, we must acknowledge that we cannot generalise our findings to those with lower abilities. However, the results are generalisable to people with or without autism who have been, on average, successful within the education system.

## Conclusion

To conclude, cis-gender autistic men and women interpret the ASA-A similarly, as do cis-gender non-autistic men and women. Autistic and non-autistic individuals do not interpret the ASA-A in the same way though. A bifactor model was supported, although specific factors captured variance in the ASA-A total score, the composite reliability indicates that the measure is essentially univocal, endorsing use of a unidimensional total score. Together, this suggests that the General Anxiety factor (encompassing the ASA-A total score) is unlikely to represent the same experience of anxiety for autistic and non-autistic individuals. Future research should explore the role of alternative gender identities and culture on presentation of anxiety. The existing cut-off can be used for autistic men and women, and group comparisons showed that women had higher anxiety scores than men in this group. Given the questions around suitability of the ASA-A for non-autistic individuals, the cut-off was not verified in this group, and it should not be applied for non-autistic people. While further item selection could lead to a three-factor solution that is invariant across autistic and non-autistic groups, it may be more appropriate to use the optimal measure for each group when comparisons are needed. Comparisons of these specific factors demonstrated that Uncertainty provided the highest relative contribution in the autistic group, and the lowest contribution in the non-autistic group, which could guide individualised interventions for anxiety in future.

## Electronic supplementary material

Below is the link to the electronic supplementary material.

Supplementary Material 1

## Electronic supplementary material

Below is the link to the electronic supplementary material.

Supplementary Material 2

## Data Availability

The data that support the findings of this study are available on reasonable request from author SC. The data are not publicly available due to containing information that could compromise the privacy of research participants.

## Footnotes

1 Data from selected measures were used for the purpose of this study. For complete methodological details, please refer to (Cassidy et al., 2021).
