# Supplementary material for: A Measurement Invariance Analysis of the Anxiety Scale for Autism–Adults in a Sample of Autistic and Non-Autistic Men and Women
Source: J Autism Dev Disord. 2024 May 14;55(3):981–96. doi: 10.1007/s10803-024-06260-2 (PMC11828802; doi:10.1007/s10803-024-06260-2)
Supplement: Supplementary file 1 — Supplementary Material 1 [file 10803_2024_6260_MOESM1_ESM.docx]

Supplementary Table 1. Frequency (%) of reported country of residence for autistic and non-autistic participants (N=658).

|  | Autistic Participants (N= 342) | Non-Autistic Participants (N= 316) |
| --- | --- | --- |
| Albania |  | 1 (0.3) |
| Australia | 4 (1.2) | 4 (1.3) |
| Belgium |  | 1 (0.3) |
| Brazil | 2 (0.6) | 1 (0.3) |
| Canada | 6 (1.8) | 6 (1.9) |
| Cyprus |  | 1 (0.3) |
| Czech Republic | 1 (0.3) |  |
| Dominican Republic | 1 (0.3) | 1 (0.3) |
| Finland | 1 (0.3) | 3 (0.9) |
| France | 1 (0.3) |  |
| Germany | 6 (1.8) | 6 (1.9) |
| Hong Kong |  | 1 (0.3) |
| Indonesia |  | 2 (0.6) |
| Ireland | 3 (0.9) | 4 (1.3) |
| Japan | 2 (0.6) |  |
| Malaysia | 1 (0.3) |  |
| Mexico |  | 1 (0.3) |
| Netherlands | 5 (1.5) |  |
| New Zealand | 1 (0.3) | 3 (0.9) |
| Norway | 1 (0.3) |  |
| Portugal | 1 (0.3) |  |
| Saudi Arabia |  | 1 (0.3) |
| Slovenia | 1 (0.3) |  |
| Spain | 2 (0.6) |  |
| Sweden | 1 (0.3) | 2 (0.6) |
| United Kingdom | 276 (80.7) | 218 (69.0) |
| USA | 18 (5.3) | 51 (16.1) |
