# Supplementary material for: A Measurement Invariance Analysis of the Anxiety Scale for Autism–Adults in a Sample of Autistic and Non-Autistic Men and Women
Source: J Autism Dev Disord. 2024 May 14;55(3):981–96. doi: 10.1007/s10803-024-06260-2 (PMC11828802; doi:10.1007/s10803-024-06260-2)
Supplement: Supplementary file 2 — Supplementary Material 2 [file 10803_2024_6260_MOESM2_ESM.docx]

*Supplementary Table 2.* Indices of model fit model degradation for measurement invariance analyses of autistic and non-autistic adult groups.

|  | Model Fit Indices | | | | | Indices of Degradation in Model Fit | | | | | |
| --- | --- | --- | --- | --- | --- | --- | --- | --- | --- | --- | --- |
|  | χ2*/df* ratio | RMSEA | PCLOSE | CFI | TLI | SRMR | ∆χ2 | ∆DF | ∆p | ∆RMSEA | ∆CFI |
| CFA | 2.215 | 0.043 | 0.966 | 0.983 | 0.958 | 0.024 |  |  |  |  |  |
| CFA (error 15&19) | 2.800 | 0.052 | 0.250 | 0.974 | 0.967 | 0.025 |  |  |  |  |  |
| CFA (+ error 3&6) | 2.617 | 0.050 | 0.532 | 0.977 | 0.970 | 0.025 |  |  |  |  |  |
| CFA (+ error 4&5) | 2.473 | 0.047 | 0.755 | 0.979 | 0.973 | 0.024 |  |  |  |  |  |
| CFA (+ error 5&12) | 2.314 | 0.045 | 0.916 | 0.981 | 0.976 | 0.024 |  |  |  |  |  |
| CFA (+ error 6&17) | 2.215 | 0.043 | 0.966 | 0.983 | 0.978 | 0.024 |  |  |  |  |  |
| Configural | 2.008 | 0.039 | 1.000 | 0.958 | 0.947 | 0.040 |  |  |  |  |  |
| Configural (error 15&19) | 1.803 | 0.035 | 1.000 | 0.967 | 0.957 | 0.040 |  |  |  |  |  |
| Configural (+ error 3&6) | 1.707 | 0.033 | 1.000 | 0.971 | 0.962 | 0.039 |  |  |  |  |  |
| Metric* | 1.925 | 0.038 | 1.000 | 0.957 | 0.951 | 0.061 | 141.516 | 40 | <.0001 | -.0005 | -0.014 |
| Metric: F0* | 1.86 | 0.036 | 1.000 | 0.962 | 0.954 | 0.059 | 82.382 | 20 | <.0001 | -0.003 | 0.009 |
| Metric: F1* | 1.736 | 0.033 | 1.000 | 0.969 | 0.961 | 0.038 | 17.062 | 5 | .0044 | 0.000 | 0.002 |
| Metric: F2 | 1.696 | 0.033 | 1.000 | 0.970 | 0.963 | 0.040 | 11.818 | 9 | .2238 | 0.000 | 0.001 |
| Metric: F2+F3* | 1.706 | 0.033 | 1.000 | 0.969 | 0.963 | 0.040 | 25.136 | 15 | .0481 | 0.000 | 0.002 |
| Metric: F2+F0* | 1.926 | 0.038 | 1.000 | 0.958 | 0.951 | 0.060 | 120.522 | 29 | <.0001 | -0.005 | 0.013 |
| Metric: F1-F3* | 1.732 | 0.033 | 1.000 | 0.968 | 0.961 | 0.041 | 42.092 | 20 | .0027 | 0.000 | 0.003 |
| Metric: F2+F1.1 | 1.694 | 0.033 | 1.000 | 0.970 | 0.963 | 0.040 | 13.058 | 10 | .2204 | 0.000 | 0.001 |
| Metric: F2+F1.1+2 | 1.691 | 0.032 | 1.000 | 0.970 | 0.963 | 0.040 | 13.736 | 11 | .2479 | 0.001 | 0.001 |
| Metric: F2+F1.1+2+3 | 1.698 | 0.033 | 1.000 | 0.970 | 0.963 | 0.040 | 17.521 | 12 | .1310 | 0.000 | 0.001 |
| Metric: F2+F1.1+2+3+4* | 1.724 | 0.033 | 1.000 | 0.969 | 0.962 | 0.040 | 27.430 | 13 | .0109 | 0.000 | 0.002 |
| Metric: F2+F1.1+2+3+5 | 1.704 | 0.033 | 1.000 | 0.970 | 0.963 | 0.040 | 21.317 | 13 | .0669 | 0.000 | 0.001 |
| Metric: F2+F1's+F3.1 | 1.700 | 0.033 | 1.000 | 0.970 | 0.963 | 0.040 | 21.633 | 14 | .0865 | 0.000 | 0.001 |
| Metric: F2+F1's+F3.1+2 | 1.699 | 0.033 | 1.000 | 0.970 | 0.963 | 0.040 | 22.973 | 15 | .0847 | 0.000 | 0.001 |
| Metric: F2+F1's+F3.1+2+3 | 1.695 | 0.033 | 1.000 | 0.970 | 0.963 | 0.040 | 23.456 | 16 | .1021 | 0.000 | 0.001 |
| Metric: F2+F1's+F3.1+2+3+4* | 1.720 | 0.033 | 1.000 | 0.969 | 0.962 | 0.041 | 32.750 | 17 | .0114 | 0.000 | 0.002 |
| Metric: F2+F1's+F3.1+2+3+5 | 1.691 | 0.032 | 1.000 | 0.970 | 0.963 | 0.040 | 23.959 | 17 | .1206 | 0.001 | 0.001 |
| Metric: F2+F1's+F3.1+2+3+5+6 | 1.689 | 0.032 | 1.000 | 0.970 | 0.963 | 0.040 | 25.078 | 18 | .1228 | 0.001 | 0.001 |
| Metric: F2+F1's+F3's+F0.1* | 1.702 | 0.033 | 1.000 | 0.969 | 0.963 | 0.045 | 30.884 | 19 | .0416 | 0.000 | 0.002 |
| Metric: F2+F1's+F3's+F0.2 | 1.686 | 0.032 | 1.000 | 0.970 | 0.964 | 0.041 | 25.687 | 19 | .1391 | 0.001 | 0.001 |
| Metric: F2+F1's+F3's+F0.2+3 | 1.681 | 0.032 | 1.000 | 0.970 | 0.964 | 0.041 | 25.844 | 20 | .1710 | 0.001 | 0.001 |
| Metric: F2+F1's+F3's+F0.2+3+4* | 1.742 | 0.034 | 1.000 | 0.967 | 0.961 | 0.049 | 46.849 | 21 | .0010 | -0.001 | 0.004 |
| Metric: F2+F1's+F3's+F0.2+3+5 | 1.676 | 0.032 | 1.000 | 0.970 | 0.964 | 0.041 | 25.984 | 21 | .2071 | 0.001 | 0.001 |
| Metric: F2+F1's+F3's+F0.2+3+5+6 | 1.675 | 0.032 | 1.000 | 0.970 | 0.964 | 0.040 | 27.184 | 22 | .2043 | 0.001 | 0.001 |
| Metric: F2+F1's+F3's+F0.2+3+5+6+7 | 1.672 | 0.032 | 1.000 | 0.970 | 0.964 | 0.040 | 27.913 | 23 | .2191 | 0.001 | 0.001 |
| Metric: F2+F1's+F3's+F0.2+3+5+6+7+8* | 1.706 | 0.033 | 1.000 | 0.968 | 0.963 | 0.040 | 40.546 | 24 | .0187 | 0.000 | 0.003 |
| Metric: F2+F1's+F3's+F0.2+3+5+6+7+9 | 1.672 | 0.032 | 1.000 | 0.970 | 0.964 | 0.039 | 29.554 | 24 | .2000 | 0.001 | 0.001 |
| Metric: F2+F1's+F3's+F0.2+3+5+6+7+9+10 | 1.676 | 0.032 | 1.000 | 0.970 | 0.964 | 0.041 | 32.500 | 25 | .1440 | 0.001 | 0.001 |
| Metric: F2+F1's+F3's+F0.2+3+5+6+7+9+10+11* | 1.755 | 0.034 | 1.000 | 0.966 | 0.960 | 0.043 | 59.727 | 26 | .0002 | -0.001 | 0.005 |
| Metric: F2+F1's+F3's+F0.2+3+5+6+7+9+10+12 | 1.681 | 0.032 | 1.000 | 0.969 | 0.964 | 0.042 | 36.050 | 26 | .0908 | 0.001 | 0.002 |
| Metric: F2+F1's+F3's+F0.2+3+5+6+7+9+10+13* | 1.693 | 0.033 | 1.000 | 0.969 | 0.963 | 0.045 | 41.566 | 27 | .0363 | 0.000 | 0.002 |
| Metric: F2+F1's+F3's+F0.2+3+5+6+7+9+10+14 | 1.689 | 0.032 | 1.000 | 0.969 | 0.963 | 0.042 | 40.104 | 27 | .0501 | 0.001 | 0.002 |
| Metric: F2+F1's+F3's+F0.2+3+5+6+7+9+10+14+15* | 1.707 | 0.033 | 1.000 | 0.968 | 0.962 | 0.044 | 47.833 | 28 | .0112 | 0.000 | 0.003 |
| Metric: F2+F1's+F3's+F0.2+3+5+6+7+9+10+14+16* | 1.706 | 0.033 | 1.000 | 0.968 | 0.963 | 0.043 | 47.478 | 28 | .0122 | 0.000 | 0.003 |
| Metric: F2+F1's+F3's+F0.2+3+5+6+7+9+10+14+17* | 1.741 | 0.034 | 1.000 | 0.966 | 0.961 | 0.046 | 58.695 | 28 | .0006 | -0.001 | 0.005 |
| Metric: F2+F1's+F3's+F0.2+3+5+6+7+9+10+14+18* | 1.784 | 0.035 | 1.000 | 0.965 | 0.958 | 0.053 | 72.765 | 28 | <.0001 | -0.002 | 0.006 |
| Metric: F2+F1's+F3's+F0.2+3+5+6+7+9+10+14+19* | 1.705 | 0.033 | 1.000 | 0.968 | 0.963 | 0.043 | 47.099 | 28 | .0134 | 0.000 | 0.003 |
| Metric: F2+F1's+F3's+F0.2+3+5+6+7+9+10+14+20* | 1.695 | 0.033 | 1.000 | 0.969 | 0.963 | 0.042 | 43.739 | 28 | .0295 | 0.000 | 0.002 |

RMSEA: Root Mean Square Error of Approximation; PCLOSE: p of Close Fit; CFI: Comparative Fit Index; TLI: Tucker-Lewis Index; SRMR: Standardized Root Mean Square Residual; ∆: Change.
Note: F0: General Anxiety factor; F1: Uncertainty; F2: Anxious Arousal; F3: Social Anxiety. Where a number follows a decimal point, this indicates the item from that factor (e.g. F3.1 is the first item loading on to the Social Anxiety factor, which is Q2; see Figure 1 for question numbers in each factor).
* Significant degradation of fit from the configural model.
